# Formulation of pea-based inks with different starches to produce customized 3D printed and baked snacks

**DOI:** 10.1016/j.crfs.2026.101320

**Published:** 2026-01-22

**Authors:** Aaditya Venkatachalam, Patrick F.C. Wilms, Júlia Patón Baeza, Maarten A.I. Schutyser, Lu Zhang

**Affiliations:** Laboratory of Food Process Engineering, Wageningen University, Bornse Weilanden 9, 6708WG, PO Box 17, Wageningen, 6700AA, the Netherlands

**Keywords:** Nutritional composition, Native starch, Pre-gelled starch, Microstructure, Printability, Young's modulus

## Abstract

The technique of 3D food printing offers the exciting potential to create customized foods. The study aimed to examine how varying composition (using different starch types) affects the 3D printability of formulations and the texture of customized pea-based snacks after post-processing. Edible inks containing a mixture of insoluble pea fibre, pea protein, and different starch types (i.e., native pea starch, pre-gelled pea starch, and potato flakes) were formulated by adding water based on ingredients’ water holding capacity (WHC) and evaluated for printability. Furthermore, the printed products were baked at 175 °C for 5, 10, and 15 min to evaluate the fracture behaviour of the post-processed products. Results showed that potato flakes had the highest WHC, followed by pre-gelled pea starch and native pea starch, owing to their microstructural differences. The WHC approach proved effective in arriving at printable samples irrespective of the changes in starch type. Moreover, the range of extrudable formulations with varying ingredient concentrations was broadened when pre-gelled pea starch and potato flakes were used. Microstructural analysis of fresh inks and baked samples indicated that native starch granules partially gelatinized during baking, while all samples showed different levels of dehydration during the baking process based on changes in moisture content. Despite the physicochemical differences that exist between the starch types, fracture properties were largely controlled by baking time. The knowledge gained from this study can facilitate a systematic approach to effectively formulate personalized plant-based foods of desired quality and texture using 3D food printing.

## Highlights

• Starches with differing physiochemical properties led to printable inks.
• Varying starch-to-protein ratio affected fresh and baked samples' moisture content.
• Dry matter of baked snacks correlated with Young's modulus despite varying starches.
• Snacks with similar texture and macronutrients can be made using different starch types.

## Graphical abstract

**Figure:**
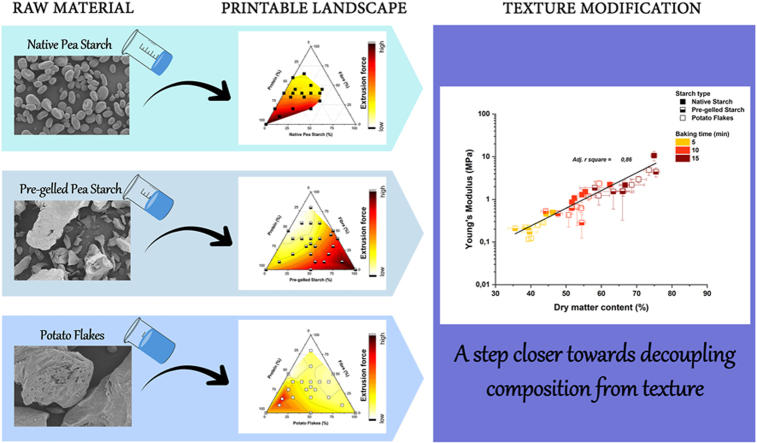

## 1 Introduction

In recent years, 3D food printing technology has been gaining popularity due to its ability to tailor foods according to individual needs, specifically in terms of nutritional needs and textural preferences. 3D printable foods should have the ability to flow through a nozzle (extrudability) and hold its shape after deposition on the printing platform (buildability) (Wilms et al., 2021). Extrudability and buildability together define the printability of a material (Venkatachalam et al., 2023; Zhu et al., 2019). The printability of materials is influenced by changes in macronutrient composition, wherein multiple researchers have detailed the importance of starch in 3D printing (He et al., 2020, 2021; Montoya et al., 2021). Starch is a complex carbohydrate present in a semi-crystalline state in nature (Villa Zabala, 2020). Researchers have explored the potential of different starch types, such as native (Venkatachalam et al., 2023) and thermally modified starches (Maniglia et al., 2020; Park et al., 2024) to develop well-printable food inks. Their results showed that thermally modified starches bind water and form a shear-thinning gel with reduced water syneresis and good printability. On the other hand, native starch was not printable by itself due to its inability to absorb water at room temperature. However, when incorporated into a mixture of ingredients, it functions as an inert filler (Venkatachalam et al., 2023). Furthermore, printable inks prepared using starch from different sources, such as cassava, corn, wheat, sweet potato, potato, and buckwheat, showed varying degrees of printability, owing to the differences in their molecular structure. In addition, the printed structures showed different textures (Ji et al., 2022). Moreover, the concentration of starch also influences printability and texture. He et al. (2020) showed the increase in potato flake concentration in a mashed potato recipe to improve printability and increase the hardness of the printed object. Thus, factors such as starch type (native or modified), source, and concentration play a crucial role in formulating well-printable inks for 3D food printing.

Printable formulations are usually made by mixing a raw material with a plasticizer, such as water, until the desired printability is achieved. Formulating a well-printable ink is not a trivial task, especially when factors such as starch type, source, and concentration come into play. Previous researchers made starch-based inks by mixing a known quantity of starch with water and heating that mixture to form a gel (He et al., 2020; Ji et al., 2022). The formed gel can be 3D-printed, which often has a high moisture content. If the source and concentration of starch need to be varied, which may be related to consumers' preferences or the availability of ingredients, extensive trial-and-error might be required before a printable formulation is achieved. A possible way to formulate printable inks with limited trial-and-error is the previously reported water-holding capacity (WHC) approach (Venkatachalam et al., 2023). In this approach, firstly, the WHC of individual raw materials (i.e., insoluble pea fibre, pea protein isolate, and native pea starch) was measured. Subsequently, a fraction of the measured WHC was added as water to formulate printable inks varying in macronutrient composition. In the previous study, only native pea starch was used to prepare formulations using the proposed WHC approach. Since native pea starch acts as an inert filler within the matrix, it does not actively contribute to the printable landscape. Therefore, it is of interest to use different types of starch with varying functionality, for instance, pre-gelatinized starches, to study the resulting printability landscape. Furthermore, when different starch types are used, it is of interest to investigate whether (i) the WHC approach can still be effectively used to formulate 3D printable inks, thereby validating this approach; (ii) the printable landscape changes while keeping the nutritional composition constant.

After formulating 3D printable inks, multiple studies measured the texture of these freshly printed high moisture content inks/gels and found textural differences in terms of hardness, springiness, and cohesiveness (Ji et al., 2022; Wang et al., 2024). However, if the consumer desires a crispy or crunchy snack, a post-processing step such as baking or frying of the printed product is necessary to significantly reduce the moisture content of the product to achieve the desired texture. Our previous study has shown that the concentration of native starch influences the texture of baked samples to a relatively small extent compared to baking time, despite the gelatinization that occurs (Venkatachalam et al., 2025). The observations of this study were based on a single starch type (native pea starch). Previous studies showing the effect of starch type on the texture of post-processed 3D printed foods are limited. So, this raised the question of whether the baking time would still largely control texture if the starch type, source, and concentration were varied. If so, this suggests that desired textures can be achieved mainly through processing conditions, irrespective of changes in composition. This would allow for flexibility in ingredient choices based on their availability.

Therefore, the aim of this study was to investigate the effect of varying composition and starch type (native pea starch, pre-gelled pea starch, and potato flakes) on 3D printability of formulated inks and the textural properties of post-processed pea-based snacks. Pre-gelled pea starch and potato flakes were chosen to compare how modification to native pea starch and how starch from a different source would influence printability, respectively. For this, properties of the starch ingredients (native pea starch, pre-gelled pea starch, and potato flakes) were characterized through composition, microstructure, and WHC. Then, edible inks containing a mixture of insoluble pea fibre, pea protein isolate, and one of the starch types in varying concentrations were formulated using a quantitative WHC approach. The edible inks were evaluated in terms of extrudability. Furthermore, the formulations containing a fixed amount of insoluble pea fibre and varying amounts of pea protein isolate, starch, and water were short-listed per starch type. The short-listed formulations were printed as hollow cylinders and baked at 175 °C for 5, 10, and 15 min. The microstructure of both fresh inks and printed baked samples per starch type was visualized using cryo-SEM. Finally, the post-processed samples were evaluated in terms of fracture properties (fracture stress and Young's modulus) and dry matter content to infer textural differences per starch type. This study provides a systematic approach to effectively formulate and process 3D printed products containing starch without compromising product quality in terms of nutritional composition, printability, and texture.

## 2 Materials and methods

Five dry ingredients, namely insoluble pea fibre, pea protein isolate, native pea starch, pre-gelatinized pea starch, and potato flakes were used in this study. The pea-based ingredients were provided by Cosucra Groupe (Warcoing SA, Belgium) with the trade names Swelite™ C Pea Fiber, Pisane™ M9 Pea Protein, Nastar™ Native Pea Starch, and Nastar™ Instant Pre-cooked Pea Starch. Dehydrated Potato Flakes H520 was kindly provided by Lamb Weston (Lamb Weston EMEA, Kruiningen, the Netherlands).

### 2.1 Raw material characterization

#### 2.1.1 Compositional analysis

The compositional analysis of raw materials was measured by Agrolab Lufa GmbH (Kiel, Germany) using the German Food and Feed Code (LFGB), similar to the methods described by Veser et al. (2024). Crude protein content (N × 6.25) was analyzed using a Kjeldhal method (LFGB, 2013; §64 L 17.00–15:, 2013-08), total dietary fibre was measured using an enzymatic gravimetric procedure (LFGB, 2017; §64 L 00.00–18:, 2017-10), crude fat and ash content were determined using the Weibull-Stoldt method (LFGB, 2017; §64 L 17.00–4:, 2017-10) and by incinerating at 550 °C (LFGB, 2002; §64 L 17.00–3:, 2002-12), respectively. The moisture content was determined using a hot air oven (LFGB, 2002; §64 L 17.00–1:, 2002-12). Carbohydrates (excluding fibre) were calculated using a difference method where the total dry weight of all the measured components in the raw materials was subtracted from 100. The calculated amount of carbohydrates mostly consisted of starch (verified using Total Starch Assay kit- Supplementary A.1). Therefore, all the carbohydrates are considered to be starch in this study. The measured composition of the raw materials used in this study is presented in Table 1.

**Table 1:** Composition of the raw materials used in this study. The star (∗) indicates the percentage displayed on a wet basis, while all the other percentages are presented on a dry matter basis.

Ingredient	Protein (%)	Starch (%)	Fibre (%)	Fat (%)	Ash (%)	Moisture (%∗)
Native Pea Starch	N/A	98.4	1.6	N/A	N/A	13.9
Pre-gelled Pea Starch	N/A	95.4	4.6	N/A	N/A	5.6
Potato Flakes	8.1	79.5	8.8	0.5	3.1	7.6
Insoluble Pea Fibre	4.8	42.7	50.6	0.4	1.5	12.7
Pea Protein Isolate	83.4	N/A	1.8	8.8	6.0	6.7

#### 2.1.2 Water holding capacity (WHC)

A method adapted from Geerts et al. (2018) was used to measure WHC. In short, 2 % w/v of the dry ingredients was mixed with demineralized water at room temperature for 24h using a shaker (Heidolph Multi Reax, Germany) at 800 rpm. The dispersions were centrifuged at 10,000g (Syrvall Lynx 4000) at 20 °C for 30 min. The supernatant was carefully decanted, and the wet pellet was transferred to a petri dish, weighed, and dried at 105 °C for 24h. WHC was calculated by dividing the mass difference (g) between the wet and dried pellet (*m*_*wet_pellet*_*-m*_*dried_pellet*_) by the mass (g) of the dry ingredient *m*_*dry_ingredient*_. These measurements were triplicated.

#### 2.1.3 Scanning electron microscopy (SEM)

Raw materials containing starch, namely native pea starch, pre-gelled pea starch, potato flakes, and insoluble pea fibre were visualized to assess microstructure before hydration. Samples were fixed onto an aluminum sample stub (9.5 mm) using carbon adhesive tabs. Following this, the samples were coated with gold using a sputter-coater (Joel Smart-Coater), and SEM images were made using a JEOL JSM-7000 at 5 kV.

### 2.2 Formulation of pea-based inks

#### 2.2.1 Formulation of inks with various starch types

To satisfy people's nutritional needs, food inks varying in macronutrient composition can be formulated by adjusting the amount of water added to ensure good 3D printability. Since one of the objectives of the current study is to effectively formulate 3D printable inks despite changes in starch types, we used the previously proposed WHC approach. In this approach, the amount of water required to make well-printable formulations was calculated based on the WHC of individual ingredients (Venkatachalam et al., 2023). It should be noted that the WHC approach is a general way of approaching the problem of formulating 3D printable inks with limited trial-and-error, rather than a standard protocol.

**Fig. 1:**
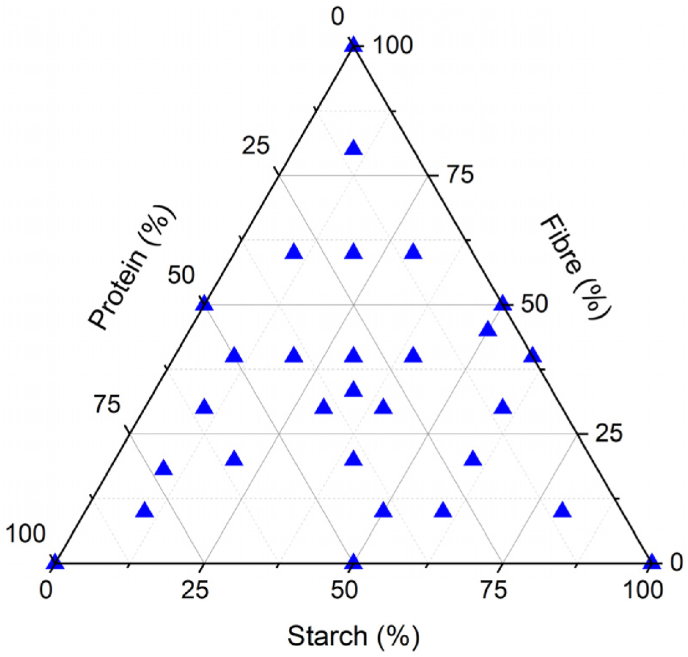
Composition of formulated edible inks as indicated by the percentage of fibre (insoluble pea fibre), protein (pea protein), and starch (either pre-gelled pea starch or potato flakes) calculated based on the total mass of ingredients (on dry matter basis).

Preliminary experiments were performed to determine the water required to make well-printable formulations. In short, equal proportions (33.33 %w/wdb) of insoluble pea fibre, pea protein isolate, and one of the starches (native pea starch, pre-gelled pea starch, or potato flakes) were mixed with varying concentrations of water (20–40 % of the individual ingredients' WHC in five levels) to form printable inks. These inks were printed as hollow cuboids (40 × 40 × 50 mm) to assess buildability (can the sample build to a height of 50 mm?) and printing precision (best visual print quality). It should be noted that this preliminary experimental procedure was based on trial-and-error to a limited extent. However, these preliminary experiments help in reducing experimental time to determine the water to be added to make printable formulations varying in macronutrient composition (Fig. 1), thereby reducing a significant portion of the trial-and-error approach. Doing so, it presents a systematic approach to achieve printable formulations even when using raw materials with different functional properties. Based on the best printable formulation per starch type using the preliminary experiments, the water required to make paste-like printable inks using native pea starch, pre-gelled pea starch, and potato flakes was estimated to be 30 %, 37 %, and 25 % of the individual ingredients’ WHC, respectively. Furthermore, multiple formulations were made by mixing water (calculated based on the WHC of individual ingredients) with varying concentrations of one of the starches, insoluble pea fibre, and pea protein isolate (Fig. 1). These formulations were tested for extrudability (section 2.2.3) as an indication of their printability to evaluate the effectiveness of the WHC approach to formulate printable inks when a different starch type is used.

#### 2.2.2 Ink preparation

The protocol for preparing inks mentioned in our previous study was used here (Venkatachalam et al., 2023). In short, the dry ingredients were weighed and roughly mixed with a spoon. Later, the dry ingredient mixture was mixed with demineralized water using a kitchen-aid mixer for 5 min (Kenwood Kitchen Mixer, UK) to obtain a paste-like dough. This was further vacuum mixed at a speed of 2000 rpm using a pressure of 30 kPa for 2 min at ambient temperature using a planetary vacuum mixer (Thinky Vacuum Mixer ARV-310LED, USA) to remove air bubbles. Parafilm was used to cover the lid of the vacuum cups to avoid moisture loss during storage. Furthermore, the inks were stored overnight in the refrigerator at 4 °C to equilibrate moisture. The following day, the inks were left at room temperature for 1h before further analysis.

#### 2.2.3 Extrusion force

The force needed to extrude inks formulated using different starches was determined by using a universal testing machine (Instron Texture Analyzer, USA), following the method developed by Venkatachalam et al. (2023). In short, the prepared inks were filled into plastic syringes (Ø = 25 mm) with a tapered nozzle (Ø = 1.5 mm) attached. A cylindrical probe (Ø = 0.5 mm) attached to a 2000N load cell was used to displace the material at a constant rate of 0.03 mm/s for 15 mm. The extrusion force was recorded, and the average equilibrium extrusion force was calculated. Measurements were performed in duplicate for 71 % of all the tested samples. The relative standard deviation of the measurements did not exceed 6.84 % with the average being 2.22 %.

#### 2.2.4 Cryo-SEM imaging of fresh inks

To understand the microstructural differences that exist after mixing the raw materials with water, a representative mixture containing fibre (40 % w/wdb), protein (20 % w/wdb), and each starch type (40 % w/wdb) was prepared as stated in section 2.2.2 and subjected to Cryo-SEM imaging. These samples are referred to as “fresh” samples hereafter. The fresh samples were mounted onto a sample holder using a thin layer of Tissue-Tek (EMS, Washington, PA, USA). Then, the samples were frozen by plunging them into liquid nitrogen. The samples were then transferred to a cryo-preparation chamber (MED 020/VCT, Leica, Vienna, Austria) where the samples were subjected to cryo-fracturing to reveal internal structure. Subsequently, the fresh samples were sputter-coated with an 8 nm layer of tungsten from three angles (90°, −45°, and +45°). Then the samples were transferred under vacuum to a field emission scanning electron microscope (Magellan 400, FEI, Eindhoven, the Netherlands) operated at a temperature of −120 °C where images were captured using a secondary electron detector at 2 kV and 13 pA.

### 2.3 3D printing and baking of pea-based snacks

#### 2.3.1 3D printing and post-processing

Well-printable inks were short-listed (based on extrusion force results) and printed using the previously mentioned extrusion force set-up i.e., plastic syringe (Ø = 25 mm) with a metal tapered nozzle (Ø = 1.5 mm) at room temperature in the form of a cylinder (Ø = 20 mm, height = 30 mm) using a custom-built printer from TNO (Prime, TNO, the Netherlands). The cylinder had an inner and outer diameter of 0.019 m^2^ and 0.027 m^2^, respectively. Using this, the inner wall cross-sectional area was calculated to be 2.5 × 10^−4^ m^2^, similar to our previous study (Venkatachalam et al., 2025). The printing speed (8.3 mm/s), layer height (0.75 mm), extrusion multiplier (1.5), retraction (0), and wall thickness (2.25 mm) were kept constant. Within 10 min after printing, the printed samples were baked at 175 °C for 5, 10, and 15 min using a convection oven (Leventi Bakermat Mastermind, Levens Middleby, the Netherlands). The baked samples were cooled for at least 30 min before measuring fracture properties (2.3.2) and dry matter content (2.3.3).

#### 2.3.2 Fracture property analysis

A texture analyzer (TA.XT plus, Stable Microsystems, UK) with a 30 kg load cell and P75 compression plate was used to compress printed samples to 80 % of their original height using a single compression method at room temperature (Venkatachalam et al., 2025). A trigger force, pre-test, and test speed of 0.49N, 2 mm/s, and 1 mm/s, respectively, were used. The force required to fracture the material was captured at constant displacement. Using the force values, fracture stress was calculated as the first peak force divided by the wall cross-sectional area of the cylinder. Young's modulus was calculated using the initial stress-strain curve in the linear region. Young's modulus was calculated between 2 % and 7 % strain. In cases where the samples collapsed before 7 % strain, calculations were made until the point where the slope equals zero. Measurements were performed five times using samples produced with the technical replicates of the same ink composition and baking conditions.

#### 2.3.3 Dry matter content

The moisture content of samples was measured by drying 2.000g of the ingredients in a petri dish for 24h at 105 °C. The moisture content (%) was calculated by dividing the mass difference (g) between the initial and dried weight of the samples (m_initial_-m_dried_) by the initial mass of the sample m_initial_ (g). Dry matter content (%) was calculated using the moisture content. Measurements were performed five times per sample.

#### 2.3.4 SEM imaging of baked samples

To visualize microstructural changes after baking, the samples used to visualize microstructure for fresh inks were printed and baked for 15 min as stated in section 2.3.1. The baked samples were mounted onto an aluminum SEM stub using conductive carbon adhesive tabs (EMS, Washington, PA, USA) and sputter-coated with approx. 12 nm layer of tungsten from three angles (90°, −45°, and +45°). Similar to the fresh inks, images were made using a field emission scanning electron microscope (Magellan 400, FEI, Eindhoven, the Netherlands) using a secondary electron detector at 2 kV and 13 pA.

### 2.4 Statistical analysis

Graphs were made using Origin (OriginPro, 2022b; OriginLab Corporation, USA). All results reported in this article are shown as mean ± SD (standard deviation). In ternary plots, only mean values are reported. ANOVA with significance levels p < 0.05 was performed to identify the effect of starch type, ratio of starch-to-protein, and baking time on Young's modulus and dry matter content using a Python script (PyCharm 2021.3.1 (Community Edition)).

## 3 Results and discussion

### 3.1 Characterization of starch-containing ingredients

The starch-containing raw materials were characterized in terms of WHC and microstructure to better understand their behavior in the formulation of edible inks (section 3.1.1) and their thermal behavior (refer to Supplementary A.2) during baking. Insoluble pea fibre was also characterized since it contained starch (42.7 % w/w db).

#### 3.1.1 Water holding capacity (WHC) and microstructure

As indicated by the measured WHC, native pea starch granules can hold the least amount of water, followed by pre-gelled pea starch, insoluble pea fibre, and potato flakes (Table 2). The measured WHC indicates the extent to which a material can trap and bind water after applying an external centrifugal force. The WHC of these raw materials can be explained by their microstructural differences.

**Table 2:**
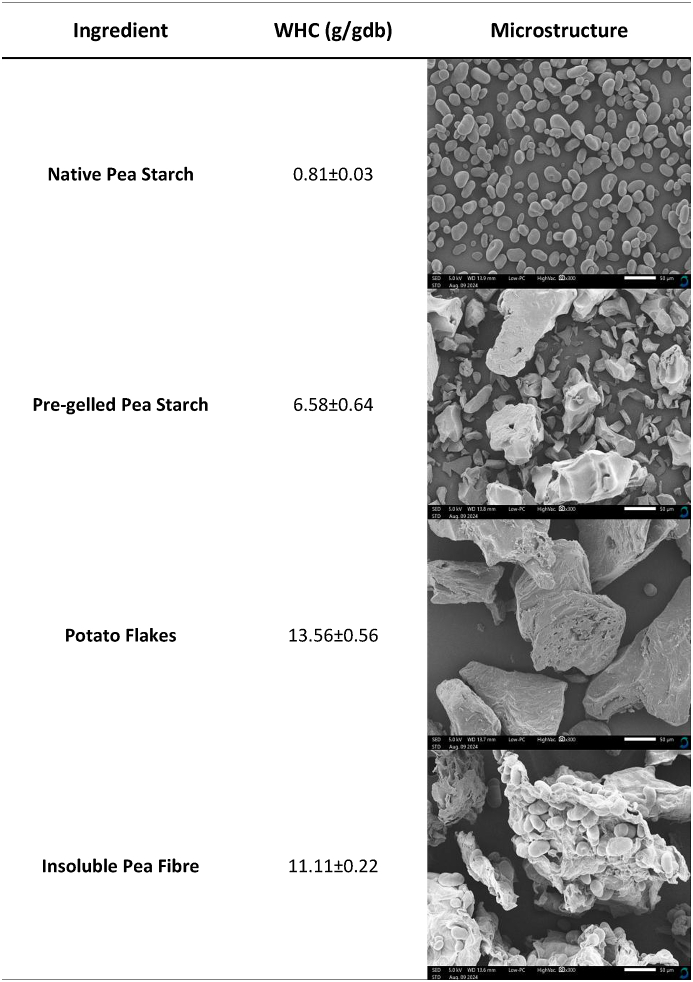
Characterization of water holding capacity (WHC) and microstructure of the different sources of starches used in this study (white scale bar in SEM images indicates 50 μm).

Native pea starch granules are small, ovoid-shaped, and smooth-surfaced (Table 2). This observation is similar to that of Zhou et al. (2019) on native field pea starch. Native starch granules are generally present in a semi-crystalline state (Liu et al., 2018) through which water cannot easily penetrate due to the lack of availability of free hydroxyl groups (Mhaske et al., 2024; Wootton and Bamunuarachchi, 1978). When water is added to native starch granules, a capillary suspension is formed where starch granules (hydrophobic particles) are suspended in water (hydrophilic liquid) (Han et al., 2024). By centrifuging and decanting, clusters of starch granules with small volumes of water occupying the voids that exist between the clusters are formed. This might explain the low WHC (0.81 ± 0.03 g/g db) of native starch. On the other hand, pre-gelled starch has larger, irregularly shaped flaky particles (Table 2). This suggests that the crystalline structure of the native starch granules is disrupted after gelatinization due to the breakage of the inter- and intramolecular hydrogen bonding (Maniglia et al., 2020). This exposes the hydrophilic hydroxyl groups, which form hydrogen bonds with water molecules (Ma et al., 2022) resulting in improved starch functionality, such as higher WHC (6.58 ± 0.64 g/g db).

Potato flakes appeared to have a larger granule size with fewer broken fragments. It also had some visible open pore-like structures on the surface (Table 2). The SEM image suggests that the potato flakes are also not in their native state, similar to pre-gelled pea starch. This is likely due to the potato flakes' preparation method, which includes cooking, mashing, and drum drying the potatoes (Lamberti et al., 2004). The authors suggest that in addition to the breakage of inter- and intramolecular bonds of the starch granules, the potato flake production process would also result in the breakage of the starch granules' cell walls, causing the starch to occupy the extracellular spaces. Such extracellular starch granules can swell more since their swelling is not constricted to a defined cellular space (Lamberti et al., 2004; Svegmark and Hermansson, 1991). This phenomenon explains the higher WHC of potato flakes (13.56 ± 0.56 g/g db). Lastly, the SEM image proved the impure nature of insoluble pea fibre. The fibre fibrils appeared to be clustered with native starch granules. However, the WHC was high (11.11 ± 0.22 g/g db) since the fibre fibrils are more porous (Lu et al., 2023) and absorb water through hydrogen bonding (Boulos et al., 2000; Hearle, 1962; Venkatachalam et al., 2023). The differences in terms of WHC and microstructure of the ingredients are expected to affect the material properties of edible inks formulated using these ingredients, which will be discussed in later sections.

### 3.2 Formulating fresh 3D printable inks using different starch types

#### 3.2.1 Identifying the printable area

The rheological behavior of an edible ink can be assessed by measuring the extrusion force (Venkatachalam et al., 2023). If the extrusion force is assessed for various ink formulations from the same ingredients, then a printable area can be defined. Therefore, in this study, the printable area for three starch-rich ingredients (native pea starch, pre-gelled pea starch, and potato flakes) were identified.

**Fig. 2:**
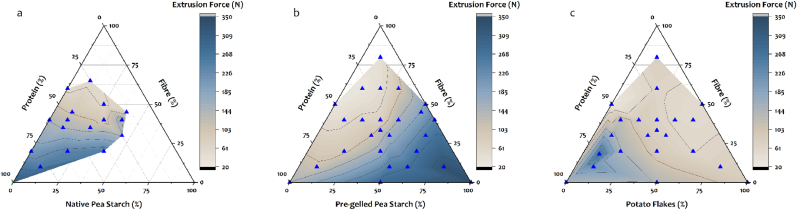
Ternary plots showing the extrusion force values of a mixture of insoluble pea fibre, pea protein, and (a) native pea starch (n = 1); (b) pre-gelled starch (n = 2); (c) potato flakes (n = 2). The blue triangles depict the measured formulations varying in macronutrient composition.

For native starch granules, 30 % of the ingredients’ WHC was added as water (since native starch has a low WHC). In doing so, the printability region obtained was relatively small (Fig. 2a). This small printable region was a result of i) samples containing a higher amount of fibre showing phase separation and ii) samples containing a higher amount of starch, that cannot form coherent inks due to the limited ability of these materials to hold water (section 3.1.1). However, in samples varying in protein, a linear increase in extrusion force with an increase in protein content was observed (Fig. 2a). This is because protein makes up the continuous phase of the matrix in which fibre and native starch granules are dispersed (based on CLSM images reported by Venkatachalam et al. (2023)). Thus, with an increase in protein content, the viscosity of the continuous phase increases, resulting in a higher extrusion force. Conversely, with a decrease in protein content, the ability of the material to form a continuous network decreases, resulting in phase separation (Venkatachalam et al., 2023).

Pre-gelled pea starch absorbs water and quickly forms a viscous gel where water is entrapped within a 3D network. Moreover, samples containing pre-gelled pea starch immobilize water more tightly compared to native starch, forming strong viscous gels due to hydrogen bonding. If too little water is used to hydrate samples containing pre-gelled starch, a highly viscous gel is formed, which leads to the printing of inconsistent broken lines. Therefore, a higher amount of water must be added to lower the gel viscosity to ensure smooth printability. In other words, the factor based on which water addition is calculated needs to be higher, hence, 37 % of the ingredients' WHC was added as water. By doing so, the extrusion force remains unchanged with an increase in protein, decreases with an increase in fibre, and increases with an increase in pre-gelled pea starch (Fig. 2b).

For potato flakes, although it has a high WHC, a lower factor (22 % of the ingredients' WHC) was required to make printable formulations based on our results from preliminary experiments. This lower factor (compared to pre-gelled pea starch) was likely caused by the presence of fibres that hold more water during the WHC measurement but may not be bound as tightly as seen in pre-gelled pea starch. Therefore, to ensure well-printable samples with limited phase separation, a lower factor of the WHC was added as water. In doing so, extrusion force increased with an increase in protein and decreased with an increase in fibre and potato flakes (Fig. 2c). The latter decrease in extrusion force is because fibre and potato flakes have a reasonably high WHC. With an increase in fibre and potato flake content in the sample, the water added also increases. Therefore, the viscosity decreases resulting in lower extrusion force values.

A major difference exists in the results obtained between the formulations prepared using native pea starch and those of pre-gelled pea starch and potato flakes. The latter ingredients are seen to result in a broader printable landscape compared to native pea starch (Fig. 2). This is because native pea starch does not possess the ability to interact with other ingredients and water and therefore cannot form the continuous phase of a formulation at room temperature (Venkatachalam et al., 2023, 2025). However, pre-gelled pea starch and potato flakes have the ability to immobilize water and form network-like structures, which makes them printable even at 100 % w/wdb starch concentrations. Moreover, the ability of pre-gelled pea starch and potato flakes to immobilize water and form network structures also allows samples with higher fibre content (lower protein content) to be extrudable without visible phase separation (Fig. 2b and c). Overall, these results show that the WHC approach can effectively formulate well-printable samples with different starch types.

#### 3.2.2 Short-listing well-printable inks

**Fig. 3:**
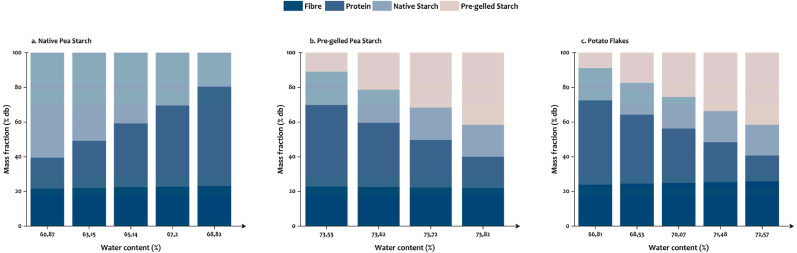
Calculated mass fractions of macronutrients (i.e. fibre, protein, and starch) in formulated inks prepared by mixing insoluble pea fibre, pea protein isolate with (a) native pea starch, (b) pre-gelled pea starch, or (c) potato flakes, in relation to the changes in the water content of food inks.

Formulated inks containing a fixed fibre content (40 % w/wdb) but varying in starch-to-protein ratios (similar to Venkatachalam et al., 2025) were short-listed for 3D printing and baking processes, to produce samples for texture analysis. The short-listed inks differed in water content and macronutrient composition, i.e., protein (10–60 % w/wdb) and starch (0–50 % w/wdb) content. Based on composition analysis, it is known that insoluble pea fibre contains a considerable amount of native starch granules (Table 1, Table 2). Therefore, the compositions were corrected for the amount of native starch present in insoluble pea fibre. Native starch and pre-gelled starch are expected to show different behaviors during baking; thus, their amounts are depicted separately. Since the concentration of pea fibre was kept constant for the short-listed inks, the amount of native starch present in the samples made using pre-gelled starch and potato flakes is also constant (Fig. 3). We will focus our discussions on the effect of starch-to-protein ratio on fresh inks and baked samples, with the consideration that the changing ratio between native starch-to-pre-gelled starch in both pre-gelled starch and potato flakes may also contribute to changes in sample properties (Fig. 3b and c, respectively).

As previously mentioned in the materials and methods (section 2.2.1), 30 %, 37 %, and 25 % of the individual ingredients’ WHC was used to prepare formulations containing native pea starch, pre-gelled pea starch, and potato flakes, respectively. The 30 %, 37 %, and 25 % for native pea starch, pre-gelled pea starch, and potato flakes translate to a final water content (on a wet basis) of 60.9–67.2 %, 73.5–73.9 %, and 66.8–71.5 % of the inks, respectively. Fig. 3 shows that the final water content of the inks was affected by the “corrected” macronutrient compositions, indicating their high dependency on the WHC of each component. For inks formulated with native pea starch (which has a low WHC), adding higher amounts of native starch (i.e., less protein) required less water to make printable formulations (Fig. 3a). Contrarily, when native starch and fibre were kept constant, the changes in the concentration of pre-gelled pea starch and protein did not result in drastic changes in the final water content of the inks (ranging between 73.5 and 73.8 %) (Fig. 3b). Since the WHC of pre-gelled starch (6.58 ± 0.64) and protein (6.17 ± 0.09) were similar, an increase in pre-gelled starch balanced out the effect of the decrease in protein in these short-listed inks, resulting in a similar water content. However, the changes in macronutrient composition of inks formulated with potato flakes did result in a slight change in water content (66.8–72.8 %) (Fig. 3c). This can be attributed to the high WHC of potato flakes.

In summary, the changes in macronutrient composition and water content of the inks are expected to influence the printing, post-processing, and the final texture of the 3D printed snacks.

### 3.3 Microstructure of fresh inks and baked snacks

**Fig. 4:**
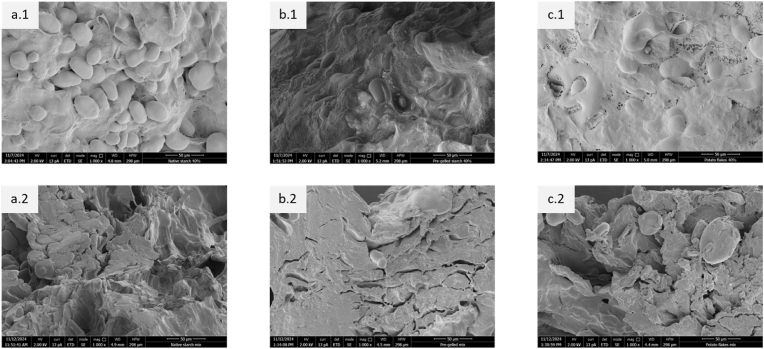
SEM images of (1) fresh and (2) baked (15 min) samples containing 40 % w/wdb insoluble pea fibre, 20 % w/wdb pea protein and 40 % w/wdb (a) native pea starch, (b) pre-gelled pea starch, and (c) potato flakes, respectively.

The microstructure of 3D printable inks and post-processed snacks can explain how the raw materials behave when hydrated with water and baked. For this, SEM images of fresh and baked mixed starch systems containing fibre (40 % w/wdb), protein (20 % w/wdb), and starch (40 % w/wdb) were visualized (Fig. 4). These percentages referred here are based on the dry matter content of the ingredients used to formulate the inks (Fig. 1). In fresh samples made with native starch, the starch granules appeared to be dispersed as a filler within the matrix (Fig. 4a1) due to their limited ability to absorb water and form a continuous network, as discussed earlier. These results align with our previous CLSM observations (Venkatachalam et al., 2023, 2025). However, upon baking, a part of the native starch granules appears to be deformed and partly form network-like structures, indicating partial gelatinization (Fig. 4a2). The presence of some remaining intact starch granules shown in Fig. 4a2 indicates partial gelatinization of starch in the matrix due to an insufficient amount of water. This incomplete gelatinization is likely caused by water redistribution among ingredients (van Rooyen et al., 2022) during storage of the inks and water removal during baking (Martínez and Gómez, 2017).

In comparison with native starch, pre-gelled starch granules in combination with fibre and protein appeared to form a more uniform and coherent gel network with uneven surfaces (Fig. 4b1). This is because pre-gelled starch contains more amylose, which has more reaction sites for the water to interact with the free hydroxyl groups (Xu et al., 2021, 2022). This mechanism of water binding results in a larger degree of water immobilization compared to samples with native starch (Supplementary A.3). When this mixture containing pre-gelled starch was printed and baked, it could be seen that the surface had multiple cracks (Fig. 4b2) (additional images- Supplementary A.4). This can be explained by water absorption and moisture release behavior in pre-gelled starch. Pre-gelled starch can retain more water compared to native pea starch (section 3.1). This means that when pre-gelled starch is added to a system, there is more water bound by the starch matrix, reducing the water availability in the surrounding environment (Laguna et al., 2011). During baking, it is plausible that water within the coherent gel matrix transforms to water vapor, resulting in an internal pressure build-up, causing the matrix to expand. The matrix cracks when the internal pressure is high, causing the water vapor to escape.

When the mixture containing potato flakes was examined with SEM, a smooth surface containing small visible pores in combination with native starch granules was observed (Fig. 4c1). During baking, this sample containing potato flakes appeared to primarily dehydrate resulting in a dense structure with a few visible cracks and pores on its surface (Fig. 4c2). The thermal expansion due to internal pressure build-up as reported for pre-gelled starch might have caused these visible pores and cracks.

Overall, there are qualitative visual differences in microstructure between the three different types of starch in both fresh and baked samples due to the difference in water binding properties of the ingredients. Future studies could benefit from quantitative image analysis of the crack length or porosity allowing for comparison of data across multiple compositions, thereby generating deeper insights. Quantitative image analysis, however, was out of scope in the current study.

### 3.4 Fracture properties of 3D printed baked snacks

#### 3.4.1 Effect of starch concentration on fracture properties

Samples made using native pea starch will partially gelatinize and dehydrate during the baking process, whereas samples made using pre-gelled pea starch and potato flakes will only dehydrate during the baking process. It is expected that these microstructural changes caused by baking the three starch types used in this study will influence the fracture properties of 3D printed pea-based baked snacks. To understand the textural differences caused by varying starch concentration, the ratio of starch-to-protein was varied, and fracture properties (fracture stress and Young's modulus) were measured for all 3D printed and baked samples per starch type. It was seen that both these fracture properties were linearly correlated with each other (r^2^ = 0.93) (Supplementary A.5). Therefore, for simplification of the data interpretation, hereafter, only Young's modulus will be discussed in detail.

**Fig. 5:**
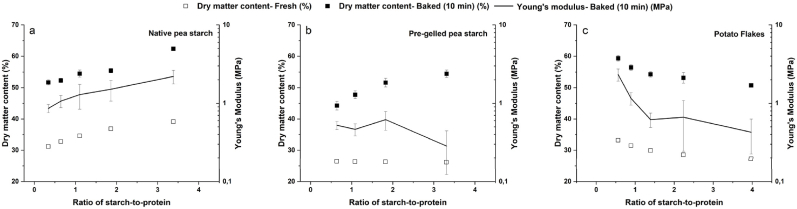
Effect of varying ratio of starch-to-protein on dry matter content of fresh and baked samples and Young's modulus of baked samples made using (a) native pea starch, (b) pre-gelled pea starch, and (c) potato flakes. Mean value of n = 5 samples are plotted in these XY plots.

The effect of varying ratio of starch-to-protein on dry matter content of fresh and baked samples (10 min) and Young's modulus of the baked snacks is visualized (Fig. 5). The ratio of starch-to-protein is calculated based on the corrected macronutrient composition of the shortlisted inks (Fig. 3). The dry matter content of the fresh samples is related to the water content of the short-listed samples, as discussed in section 3.2.2. Therefore, in this section, the changes in dry matter content after baking will be discussed.

For inks formulated with native starch and potato flakes, the average moisture loss during baking was similar, despite the changes in starch-to-protein ratio (Fig. 5a & c). However, for inks formulated with pre-gelled starch, a higher starch-to-protein ratio resulted in a higher final dry matter content of the baked snacks, although the initial dry matter of the fresh inks was similar (Fig. 5b). This difference might be explained by crack formation during baking, which creates more surface area for moisture removal (Fig. 4b2).

Our results showed that an increase in the starch-to-protein ratio resulted in an increase in Young's modulus for samples formulated with native starch (Fig. 5a). This relationship was opposite for potato flakes (Fig. 5c). These changes in Young's modulus can be primarily associated with the WHC of native starch and potato flakes (section 3.1). The higher the amount of native starch in an ink, the lower the amount of water added to formulate the ink, whereas this trend was the opposite for potato flakes. Moreover, in native starch, the partial gelatinization of starch can lead to additional hydrogen bonding (Tako et al., 2014) which may also contribute to the increase in Young's modulus (based on the SEM image, Fig. 4a 0.1). Similarly, at lower potato flake concentrations, a significant amount of native starch from the fibre (Fig. 3c) can undergo partial gelatinization, resulting in higher Young's modulus values (Fig. 5c). In pre-gelled starch, despite the increase in dry matter content, with an increase in the ratio of starch-to-protein, Young's modulus did not show a significant difference except for the sample containing the highest pre-gelled starch (a starch-to-protein ratio of 3.31), which showed a decreasing trend. The latter observation could be due to multiple cracks observed on the surface of this particular sample (with a ratio of starch-to-protein of 3.31 and its microstructure can be seen in Fig. 4b2). When a force is applied to an object with pre-existing cracks, small cracks propagate to larger cracks to reduce the total energy of the system until the material fractures (Griffith, 1921). The higher the number of cracks, the quicker the material collapses upon compression. This may explain why this particular sample had the lowest Young's modulus. Moreover, even though there was a higher fraction of native starch (coming from the fibre) in samples containing a lower pre-gelled starch to protein ratio, the effect of this native starch on Young's modulus was not evident.

Overall, higher concentrations of native starch and lower concentrations of potato flakes appear to result in high dry matter content and high Young's modulus values after baking. Moreover, for native pea starch and potato flakes, similar to observations by Rinaldi et al. (2010); Vandenberghe et al. (2014) dry matter content was linearly correlated with Young's modulus. Conversely, in pre-gelled starch, with an increase in dry matter content after baking, the Young's modulus did not appear to differ significantly, except for the highest ratio of ratio-to-protein.

#### 3.4.2 Effect of baking time on fracture properties

**Fig. 6:**
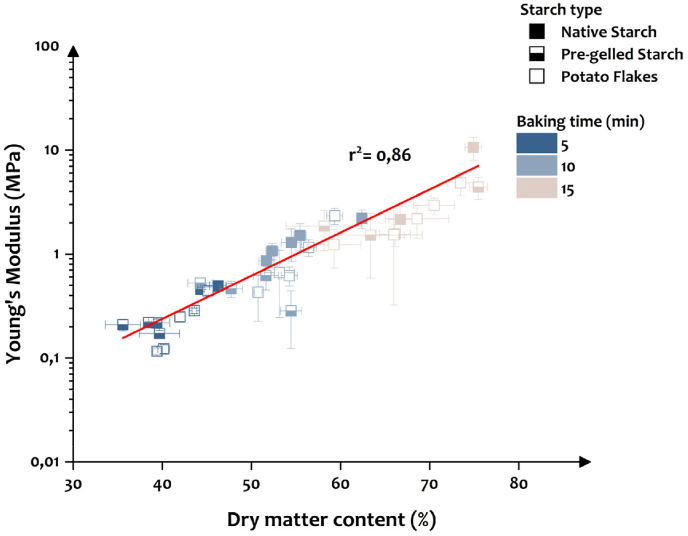
Effect of the three starch types and baking times used in this study on Young's modulus and dry matter content. Mean value of five replicates is plotted in this XY plot.

It is evident that dry matter content is connected to the hardness of the baked samples. Since dry matter content is influenced by baking time (Gulati and Datta, 2015), it is possible to estimate the baking time required to reach a target hardness or Young's modulus. The same is possible using the different starch types in this study (Fig. 6). It can be observed that dry matter content correlates linearly with Young's modulus irrespective of changes in starch type (r^2^ = 0.86). Moreover, as expected with an increase in baking time, both dry matter content and Young's modulus increased (Venkatachalam et al., 2025). Overall, from these results, when starch type, concentration, and baking time are varied systematically, the latter has a more significant effect on Young's modulus (p < 2.2·10^−16^) and dry matter content (p < 2.2·10^−16^) compared to the former two (ANOVA table listed in Supplementary A.6).

The Young's modulus values range from 0.12 MPa to 10.63 MPa with dry matter contents ranging from 35.57 % to 75.47 % (Fig. 6). These values fall within the range of our previously reported Young's modulus and dry matter content values of 0.005 MPa–20.76 MPa and 27.85 %–88.48 %, respectively (Venkatachalam et al., 2025). From our results, it is evident that dry matter content largely controls the fracture properties of the baked pea-based snacks. Overall, although there were visible differences between the three starch types used in this study (section 3.1 and supplementary A.2), these differences appear to be less significant than the effect of baking time on Young's modulus and dry matter content.

### 3.5 Starch-water interactions: Pre- and post-baking

In this study, the effect of starch type and concentration on making 3D printable formulations and the resulting textural properties achieved after post-processing were investigated. The textural properties are predominantly determined by the dry matter content (Fig. 6), with a small percentage of texture variation, i.e. 14 %, determined by other factors. Among these other factors, the ingredient composition is likely the main variable (Fig. 5) and therefore presents an opportunity to fine-tune textural properties beyond dry matter content alone. The effect of different ingredients, e.g. starch type or protein-starch ratio, is linked to 1) the amount of water that was present in the initial fresh sample, originating from the differences in WHC of starch ingredient and protein, which resulted in differences in moisture loss during baking and final moisture content of the baked sample; and 2) the type of starch ingredient used affected the extent of starch gelatinization that occurred during baking and thereby microstructure formation (Figs. 4) and 3) in specific cases the microstructure led to an increased tendency to form cracks, which not only accelerates drying, but also directly affects textural properties, i.e. decrease the Young's modulus. Based on these differences, we propose an explanatory guideline for water addition and microstructural changes of fresh inks and baked snacks when different sources of starch are used (Table 3). Doing so, the amount of water to be added to make printable formulations using different starch types, and the role of the ingredients in contributing to the texture (fracture properties) of 3D printed foods is explained.

**Table 3:**
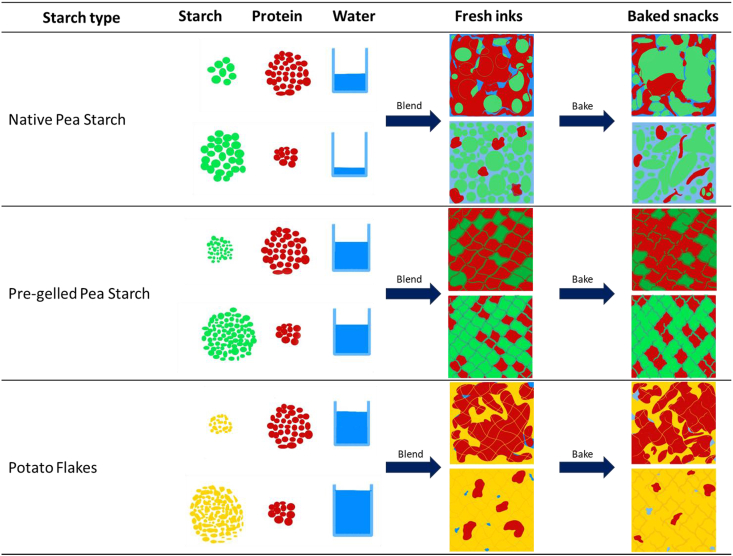
Proposed explanatory guideline for water addition and microstructural changes that occur during ink preparation and baking depending on the starch type (native pea starch, pre-gelled pea starch, and potato flakes) at low and high concentrations.

#### 3.5.1 Native starch

When the ratio of native starch-to-protein is lower, a higher amount of water is required to make a printable ink and vice-versa (Table 3). When these ingredients are blended to make a printable ink, depending on the concentration of protein, the microstructure of the fresh inks varies. When there is a higher amount of protein in the ingredient mix, the protein makes up the continuous phase of the system in which starch granules are dispersed. On the other hand, when there is less protein (20 % w/wdb) in the system, starch granules and protein are present in the dispersed phase. From our previous study, it is known that samples containing native starch up to 60 %w/wdb are printable (Venkatachalam et al., 2023). When such inks are printed and baked, the native starch granules undergo partial gelatinization. Depending on the protein concentration, upon gelatinization, the starch granules either form a discontinuous network with protein (higher protein content) or continue to remain part of the dispersed phase due to partial gelation (lower protein content). In the earlier case, samples tend to be stiffer, resulting in a higher Young's modulus, while the opposite is true for samples with lower protein content.

#### 3.5.2 Pre-gelled starch

Samples made using pre-gelled pea starch require more water to make printable inks compared to native starch. A similar amount of water is needed to make printable inks, irrespective of whether the ratio of pre-gelled starch-to-protein is low or high. This has to do with the fact that both pre-gelled starch and protein have a similar WHC. In both cases, a bicontinuous gel is formed (Table 3); however, depending on the concentration of pre-gelled starch and protein, the dominant ingredient in the bicontinuous gel would differ. If there is more pre-gelled starch, the gel would be starch-dominant. Similarly, at lower pre-gelled starch contents, the gel would be protein-dominant. In both cases, water is immobilized within the sample. However, when protein dominates the gel, it can inhibit the gelatinization of starch but form a network in which water is trapped (Yang et al., 2019). On the other hand, in a starch-dominant gel, a firm gel is formed. Upon baking at high temperatures, protein-dominant gels display more elasticity and, therefore, have a higher Young's modulus before fracture (Yang et al., 2019). Meanwhile, starch-dominant gels fracture relatively faster, resulting in lower Young's modulus values.

#### 3.5.3 Potato flakes

Similar to pre-gelled pea starch, in potato flakes, with an increase in the ratio of potato flakes-to-protein, the water added to make printable inks increases. When a lower ratio of potato flakes-to-protein is added, a bicontinuous gel in which potato flakes and protein form a bicontinuous network is formed. When the concentration of potato flakes increases, the potato flakes make up the continuous phase of the matrix in which protein is dispersed. These samples, upon baking, primarily undergo dehydration and, to a relatively smaller extent, crack during baking.

### 3.6 Implications of this work

The current study discusses two main aspects, namely: nutritional composition and textural properties of 3D printed foods. From a nutritional composition perspective, the current study shows the potential of the WHC approach to result in well-printable samples despite the changes in starch type. Particularly, pre-gelled starch and potato flakes, compared to native starch, were shown to absorb water and form network-like structures that reduced phase separation. This water-binding capacity resulted in minimal phase separation, even with higher fibre contents, thereby expanding the printability landscape. This result implies that by choosing the appropriate starch type, the printability landscape can be expanded without altering the nutritional profile of the formulation.

From a texture perspective, this study highlights the potential of extrusion-based 3D food printing to achieve the target Young's modulus of pea-based snacks despite the variation in macronutrient composition (i.e., starch-to-protein ratio). This was possible through the correlation between the two dependent variables – Young's modulus and dry matter content. The latter was controlled by changing the water added to the formulations depending on the starch type and the baking time. Despite the changes in the starch type, a similar Young's modulus was achieved per dry matter content. This means there is more freedom in terms of raw material selection, where different starch types can be used interchangeably without significantly affecting the fracture properties of the final baked product. This would also provide more flexibility in terms of material sourcing and cost optimization. It is important to highlight that fibre content was kept constant in this study, while the ratio of starch-to-protein was varied per starch type. Results from the study of Jebalia et al. (2022) stated that the presence of insoluble pea fibre might promote brittle fracture due to the creation of weak spots in pea-based extrudates. This suggests that varying insoluble pea fibre might result in varying fracture properties. Therefore, it might be worthwhile to consider the effect of insoluble pea fibre on fracture properties in future studies.

Although this study establishes that dry matter content governs the fracture properties, our results are limited to baked samples. When other post-processing methods, such as steaming, are used, the slope of the relationship between dry matter content and Young's modulus can vary (Venkatachalam et al., 2025) which would result in a different perceived stiffness by the consumer. Therefore, it is essential to further investigate the relationship between Young's modulus and dry matter content using other post-processing conditions with different modes of heat transfer to enhance the understanding, prediction, and customization of target snack textures. We've shown the potential of varying dry matter contents to achieve similar fracture properties despite the variation in starch types, which is an advantage for the customization of foods. However, studies have shown formulations containing similar dry matter content but varying in composition (Lille et al., 2020) and infill patterns (Ma et al., 2024) to result in products with different textures. This suggests that while dry matter content is crucial for achieving larger texture differences, composition and the printing settings are important factors in fine-tuning the final texture of the snack (Venkatachalam et al., 2025).

## 4 Conclusion

Developing 3D printable inks using different starch types with limited trial-and-error is a challenging task. Furthermore, achieving the desired textural properties despite variations in ink composition adds an additional layer of complexity to the process. In the current study, we showed that compared to native pea starch, an expanded printable landscape can be achieved when inks are formulated using pre-gelled pea starch and potato flakes due to their higher WHC. The WHC approach was shown to be an effective method in achieving well-printable inks using various types of starch-rich ingredients. However, the amount of water added to make printable inks varied per starch type due to the differences in how they interact with water. Moreover, changes in the macronutrient composition (specifically the ratio of starch-to-protein) affected the dry matter content of both fresh inks and baked snacks. However, by adjusting the initial water content of fresh inks and the baking time, customized textures (in terms of Young's modulus) can be achieved, irrespective of changes in macronutrient composition. These insights can be used in the future to develop personalized nutrition using macronutrient compositions from different sources. Moreover, it is a step towards designing target textures irrespective of changes in composition; in other words, it is a step towards decoupling composition from texture. Lastly, from the knowledge gained from this study, it is recommended that future research should focus on developing a mechanistic understanding of ingredient-water interactions that influence the printability of inks and texture of post-processed 3D printed foods.

## Author contributions

Aaditya Venkatachalam: Conceptualization, Methodology, Investigation, Validation, Formal Analysis, Writing-original draft preparation, Writing-review & editing. Patrick F.C. Wilms: Conceptualization, Supervision, Writing-review & editing. Júlia Patón Baeza: Investigation, Formal Analysis. Maarten A.I. Schutyser: Supervision, Writing-review & editing, Funding acquisition, Project administration. Lu Zhang: Conceptualization, Validation, Supervision, Writing-review & editing, Funding acquisition.

## Declaration of generative AI and AI-assisted technologies in the writing process

During the preparation of this work, the author(s) used Copilot in order to improve the coherence of sentences. After using this tool/service, the author(s) reviewed and edited the content as needed and take(s) full responsibility for the content of the publication.

## Declaration of competing interest

The authors declare that they have no known competing financial interests or personal relationships that could have appeared to influence the work reported in this paper.

## Data Availability

YODAData underlying the publication: Formulation of pea-based inks with different starches to produce customized 3D printed and baked snacks (Original data)
