# Supplementary material for: Formulation of pea-based inks with different starches to produce customized 3D printed and baked snacks
Source: Curr Res Food Sci. 2026 Jan 22;12:101320. doi: 10.1016/j.crfs.2026.101320 (PMC12876297; doi:10.1016/j.crfs.2026.101320)
Supplement: Multimedia component 1 [file mmc1.docx]

# **Supplementary Information**

# Formulation of pea-based inks with different starches to produce customized 3D printed and baked snacks

# A.1. Total starch content present in starch-containing samples

The starch content in the raw materials was determined using a Total Starch Assay kit from Megazyme (K-TSTA-100A, Megazyme, Ireland) following the method stated by Wetterauw et al. (2023). The starch content couldn’t be measured for native pea starch and pre-gelled pea starch due to gel formation in the samples. However, both native and pre-gelled pea starch was stated to have >99%db starch content based on the manufacturer's specifications. Moreover, the measured starch content for potato flakes and insoluble pea fibre were 73.14±2.2 and 42.41±1.75, respectively. Since, the measured total starch content was within the range of the total carbohydrates present in the raw materials, all carbohydrates were considered as starch in this study.

# A.2. Differential Scanning Calorimetry (DSC)

A differential scanning calorimeter (DSC-250, TA Instruments, USA) calibrated for heat flow and temperature using Indium (m.p. 156.6°C) was used for measuring the thermogram of each raw material used in this study. 0.1g of the raw materials were dispersed in 1g of water and hydrated for 24h. Approx. 60 mg of each sample was transferred into stainless steel high-volume DSC pans (TA Instruments, USA) to measure gelatinization enthalpy. During the DSC analysis, an empty reference pan and a sample pan were held at 10°C for 1 min and then heated to 140°C at a rate of 10°C/min. Gelatinization enthalpy (ΔH) obtained from the area of the peak was corrected based on the starch content present in the raw materials. Measurements were at least duplicated for each sample.

A.2.1. Gelatinization behavior of different starch sources

DSC was used to study the gelation behavior of the starches used in this study. A summary of the onset temperature, peak temperature, and gelatinization enthalpy (ΔH) corrected for per gram starch granules present in the samples is shown in Table A.2.1. Potato flakes and insoluble pea fibre have slightly higher onset and peak temperatures compared to native and pre-gelled pea starch. This observation can be explained by the porous nature of potato flakes and insoluble pea fibre (Lu et al., 2023). When the porosity of a material is higher, its bulk density is lower, resulting in lower thermal diffusivity (Emami et al., 2007). Moreover, the presence of protein and fibre in potato flakes and insoluble pea fibre may also decrease the thermal diffusivity, delaying the heat from penetrating the starch granules, thereby delaying the gelatinization process (Altay & Gunasekaran, 2006). The gelatinization enthalpy (ΔH) was highest for native starch granules, followed by insoluble pea fibre, pre-gelled pea starch, and potato flakes. This observation can be explained by the starch gelatinization process, during which the amylopectin crystals within the crystalline lamellae of the starch granules are broken down. The disordering and breaking down of amylopectin crystallites is represented by ΔH (Fu et al., 2012). This means that native pea starch and insoluble pea fibre have higher crystalline parts, also evident in the SEM images, resulting in higher ΔH values. Furthermore, the starches in pre-gelled pea starch and potato flakes are priorly gelatinized (depicted by SEM images in Table A.2.1), wherein there are more damaged starch granules with exposed hydrophilic sites. Such damaged starch granules have a lower gelatinization enthalpy (Fu et al., 2012; León et al., 2006).

Table A.2.1. Table showing the onset temperature, peak temperature, and the gelatinization enthalpy of different starch-containing samples used in this study.

| **Ingredient** | **Onset Temp (°C)** | **Peak Temp (°C)** | **ΔH (J/g)** |
| --- | --- | --- | --- |
| **Native Pea Starch** | 60.56±1.02 | 68.71±0.72 | 1.41±0.24 |
| **Pre-gelled Pea Starch** | 57.66±8.36 | 68.51±3.97 | 0.26±0.17 |
| **Potato Flakes** | 62.67±4.26 | 71.64±1.35 | 0.16±0.01 |
| **Insoluble Pea Fibre** | 61.73±1.77 | 71.40±2.25 | 0.98±0.2 |

Moreover, it is noteworthy that preparing a homogenous sample containing pre-gelled pea starch for DSC measurement was found difficult because the dry powder formed lumps once it was in contact with water, preventing water from diffusing further into the matrix. This resulted in relatively large standard deviations in the DSC results of pre-gelled pea starch (Table A.2.1).

# A.3. Water binding in native and pre-gelled pea starch

Time-domain nuclear magnetic resonance (TD-NMR) was used to measure the water distribution in native pea starch and pre-gelled pea starch. Samples were prepared by varying the water content between 30-80% and ingredient concentration between 20-70%, which resulted in a mass ratio between water and ingredient ranging from 0.43 to 4. The samples were loaded into NMR tubes and measured for T_2_ relaxation. Experiments were performed on Bruker NMR ND spectrometers operating at a field frequency of 20 MHz for proton using quadrature phase detection. A FID-CPMG pulse sequence was applied where the FID was acquired during the first 100 μs and the CPMG sequence with a TE of 0.2 ms and 4K pulses in total with a 4-step phase cycle and 8 averages. A dwell time of 0.4 µs was used and a recycle delay time of 5 seconds. The 90° and 180° pulse lengths were set to 2.5 µs and 4.8 µs respectively. The raw data was phase and background corrected (empty tube) with MATLAB and the CPMG part was exponentially weighed to reduce the data size to 1K points. Origin software was used to deconvolute the T_2_ NMR decay curves to obtain the phase composition distribution and T_2_ NMR relaxation values. For this, a Gaussian function was used to fit the rigid/solid fraction and several exponential functions for the more mobile/liquid fractions according to:

$$\begin{aligned} \frac{M_{z}\left( t \right)}{M_{0}}=G\times\exp\left( {-0.5\times\left( \frac{-t}{Gt} \right)}^{2} \right)+\sum_{i =1}^{n} L_{i}\times exp\left( -\frac{t}{T_{2,i}} \right)\#\left( Eq 1 \right) \end{aligned}$$

where G and L_i_ are the amplitudes of the phase contributions with different relaxation behavior and G_t_ and T_2,i_ the corresponding transversal relaxation times. Measurements were performed at 20 °C. Standard deviations of relaxation times and amplitudes were estimated from measurements performed in duplicate.

The average T2 value was calculated using the phase contributions and the relaxation times as follows:

$$\begin{aligned} Average T_{2}= \frac{\sum T_{2,i}\times L_{i}}{\sum L_{i}}\#\left( Eq 2 \right) \end{aligned}$$

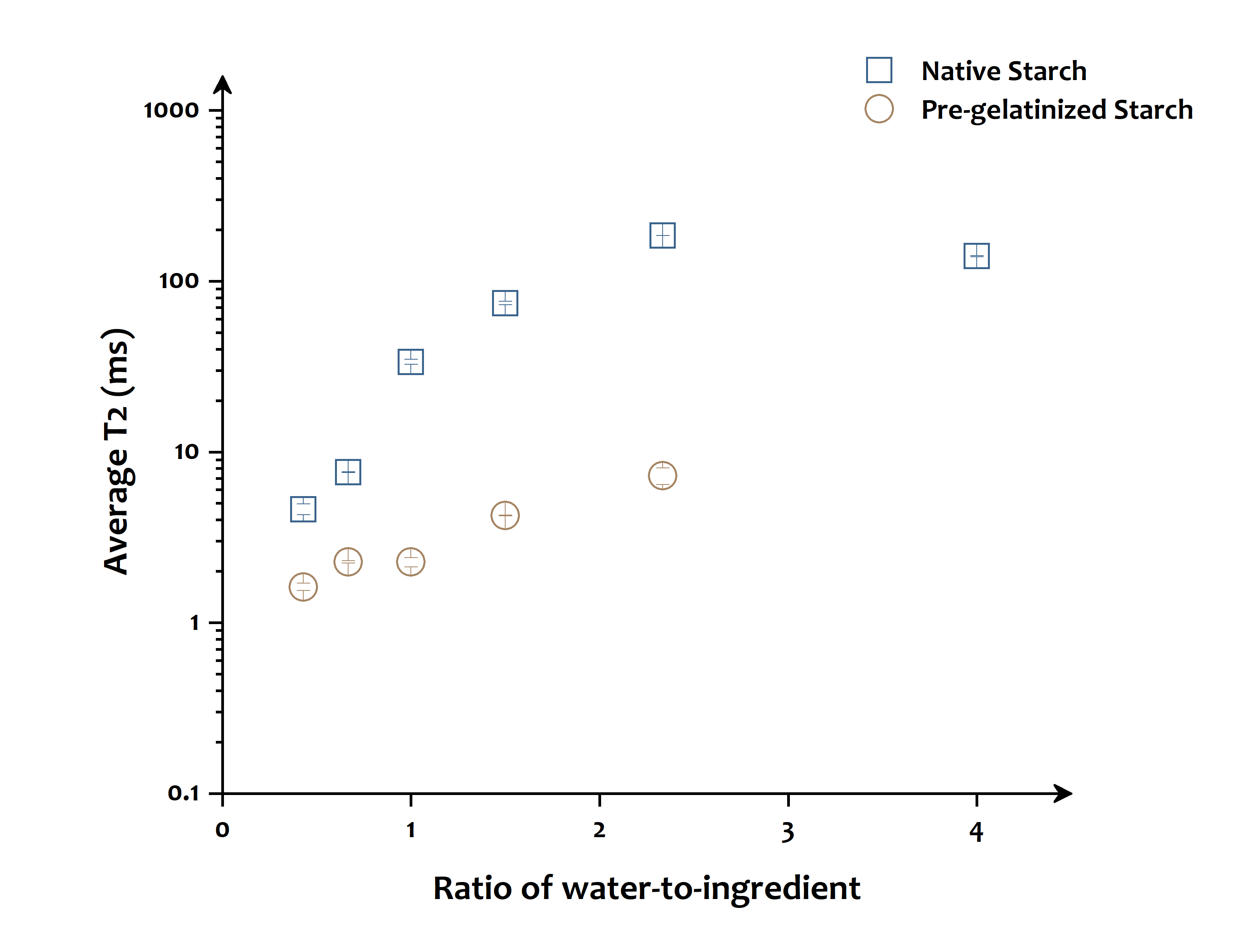
The average T2 values of native starch and pre-gelled starch were compared with each other. It can be seen that as the ratio of water increases, the average T2 increases, suggesting that as a higher amount of water is added, the water mobility increases (Fig A.3.1). When comparing the water mobility between native pea starch and pre-gelled starch, all pre-gelled starch samples have lower water mobility compared to native pea starch. This means that water is better immobilized by pre-gelled pea starch while there is a higher population of free water present in native pea starch.

Figure A.3.1. Average T2 values of native pea starch and pre-gelled pea starch with varying water-to-ingredient ratio. Relative errors are plotted as error bars.

# A.4. Additional pictures to show crack formation in baked pre-gelled starch samples


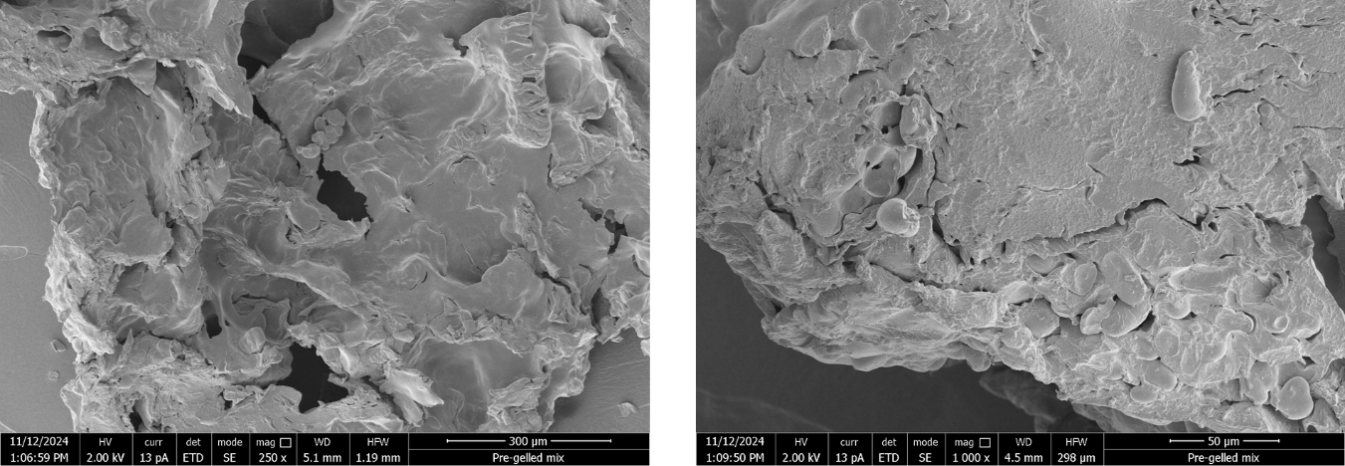


Figure A.4.1. SEM images at 300µm(left) and 50µm(right) magnification showing the cracks that exist on the surface of baked pre-gelled pea starch samples containing 40%w/wdb fibre, 20%w/wdb protein, and 40%w/wdb pre-gelled starch. This sample corresponds to a starch-to-protein ratio of 3.31.

#
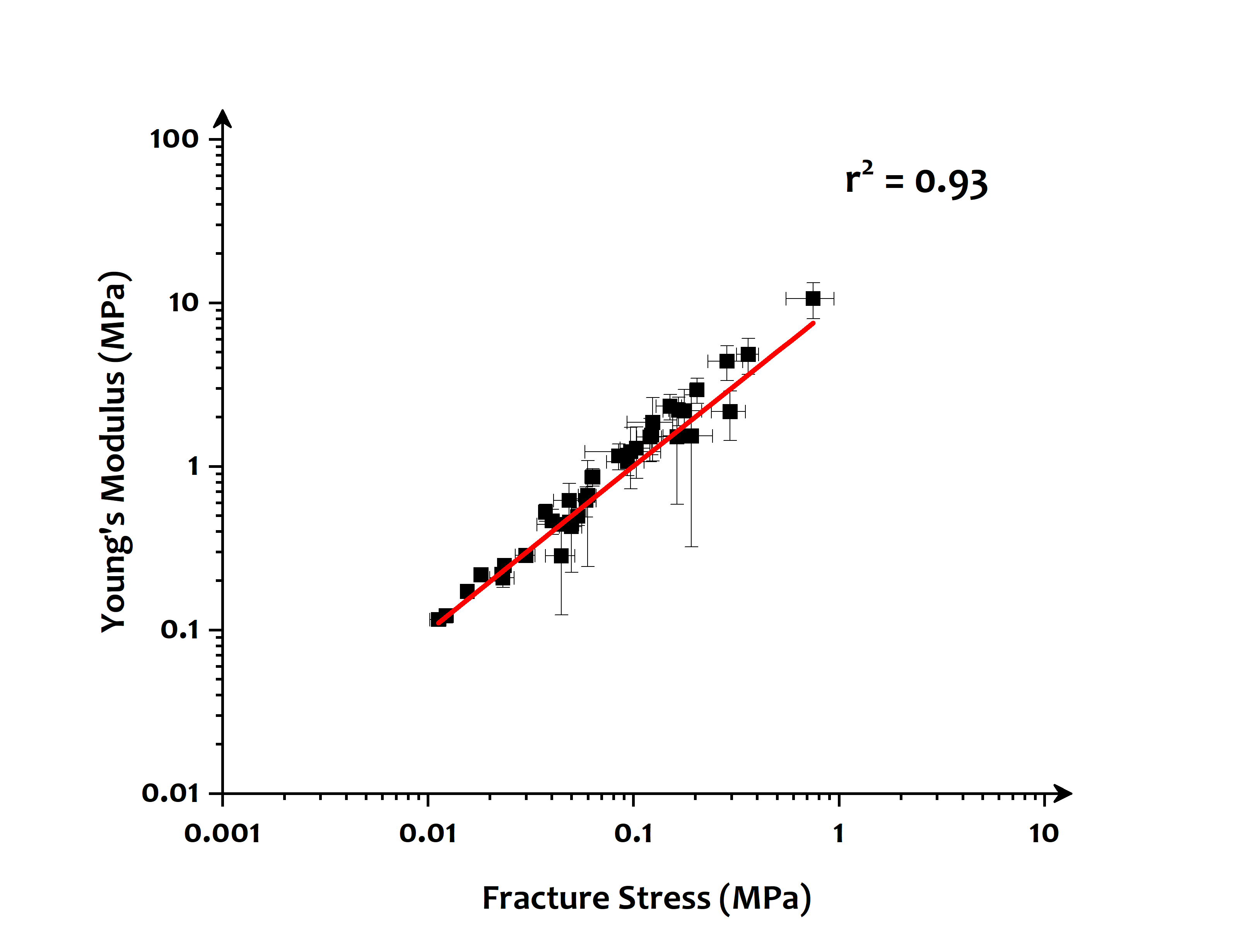
A.5. Linear relationship between fracture stress and Young’s Modulus

Figure A.5.1. XY plot showing the linear correlation that exists between fracture stress and Young’s modulus.

# A.6. Analysis of variance to investigate the effect of starch type, ratio of starch-to-protein, and baking time on Young’s modulus and dry matter content

ANOVA analysis was performed to understand the significance of the effect of the independent variables on the responses, namely: log Young’s modulus and log dry matter content. The factors, their interaction parameters, and their significance on the responses are shown in Table A.2.2. This table suggests that baking time has the highest effect on Young’s modulus, followed by water content, starch type, and ratio of starch-to-protein. Similarly, for dry matter content, baking time>water content>starch type.

Table A.2.2. ANOVA table showing model accuracy and the F-value and p-value of various factors on Young’s modulus and dry matter content. The Pr (>F) depicts the statistical significance of the factors, with *** depicting p<0.001 and ** depicting p<0.01.

|  | **Young’s Modulus** | | **Dry matter content** | |
| --- | --- | --- | --- | --- |
|  | **F-value** | **Pr (>F)** | **F-value** | **Pr (>F)** |
| **Model accuracy**  **(adj r^2^)** | 0.9198 | | 0.9202 | |
| Starch type | 21.57 | 2.14·10^-6^*** | 7.82 | 0.002** |
| Ratio of starch-to-protein | 9.56 | 0.005** | 0.70 | 0.411 |
| Water content | 42.03 | 5.04·10^-7^*** | 24.43 | 3.25·10^-5^*** |
| Baking time | 313.26 | <2.2·10^-16^*** | 368.81 | <2.2·10^-16^*** |
| Starch type·Baking time | 0.25 | 0.780 | 0.54 | 0.587 |
| *** p<0.001; ** p<0.01 | | | | |

# References

Altay, F., & Gunasekaran, S. (2006). Influence of Drying Temperature, Water Content, and Heating Rate on Gelatinization of Corn Starches. *Journal of Agricultural and Food Chemistry*, *54*(12), 4235-4245. <https://doi.org/10.1021/jf0527089>

Emami, S., Tabil, L., & Tyler, R. T. (2007). Thermal properties of chickpea flour, isolated chickpea starch, and isolated chickpea protein. *Transactions of the ASAE 50(2): 597-604*, *50*, 597-604. <https://doi.org/10.13031/2013.22648>

Fu, Z.-q., Wang, L.-j., Li, D., & Adhikari, B. (2012). Effects of partial gelatinization on structure and thermal properties of corn starch after spray drying. *Carbohydrate Polymers*, *88*(4), 1319-1325. <https://doi.org/https://doi.org/10.1016/j.carbpol.2012.02.010>

León, A., Barrera, G., Pérez, G., Ribotta, P., & Rosell, C. (2006). Effect of damaged starch levels on flour-thermal behavior and bread staling. *European Food Research and Technology*, *224*, 187-192. <https://doi.org/10.1007/s00217-006-0297-x>

Lu, Y., Zhang, L., & Schutyser, M. A. I. (2023). Linking particle morphology and functionality of colloid-milled dietary fibre concentrates from various plant sources. *Lwt*, *186*, 115206. <https://doi.org/https://doi.org/10.1016/j.lwt.2023.115206>

Wetterauw, K., Wilms, P., Tiggeloven, A., Boom, R., van der Linden, E., Venema, P., & Schutyser, M. (2023). Dry fractionation for endosperm recovery from a barley malt waste stream. *Journal of Food Engineering*, *357*, 111630. https://doi.org/https://doi.org/10.1016/j.jfoodeng.2023.111630
